# Mechanical thrombectomy using the retrograde semi-retrieval technique for patients with underlying intracranial atherosclerotic stenosis

**DOI:** 10.3389/fneur.2023.1280181

**Published:** 2024-01-12

**Authors:** Wei Wang, Yongbo Xu, Bohao Zhang, Shuling Liu, Zhenjian Ma, Sifei Wang, Pinyuan Zhang, Ming Wei

**Affiliations:** ^1^Department of Neurology, Tianjin Huanhu Hospital, Tianjin, China; ^2^Key Laboratory of Cerebral Vascular and Neurodegenerative Diseases, Tianjin Neurosurgical Institute, Tianjin, China; ^3^Clinical College of Neurology, Neurosurgery and Neurorehabilitation, Tianjin Medical University, Tianjin, China; ^4^Department of Neurosurgery, Tianjin Huanhu Hospital, Tianjin, China; ^5^Department of Neurosurgery, The Second Hospital of Tianjin Medical University, Tianjin, China; ^6^Department of Neurosurgery (Cerebrovascular Disease), The Third Hospital of Hebei Medical University, Shijiazhuang, China; ^7^Department of Academy of Medical Engineering and Translational Medicine, Tianjin University, Tianjin, China

**Keywords:** thrombectomy, acute ischemic stroke, large vessel occlusion, stent retriever, retrograde semi-retrieval technique

## Abstract

**Background:**

The retrograde semi-retrieval technique (RESET) has been described as a modified technique for endovascular thrombectomy (EVT) whose safety and efficacy for intracranial atherosclerosis stenosis (ICAS) patients remain uncertain. This article presents our single-center experience, comparing RESET vs. non-RESET in ICAS patients.

**Materials and methods:**

We analyzed 327 consecutive ICAS patients who underwent EVT at Tianjin Huanhu Hospital from January 2018 and December 2022. Patients were categorized into two groups: RESET and non-RESET. The primary outcome was the first-pass effect (FPE). Secondary outcomes included successful reperfusion, functional independence at 90 days, mortality, and symptomatic intracranial hemorrhage (sICH).

**Results:**

RESET was significantly associated with FPE [adjusted odds ratio (aOR) 2.00, 95% confidence interval (CI) 1.03–3.87, *p* = 0.040]. RESET was not significantly associated with successful reperfusion (aOR 1.5, CI 0.55–4.06, *p* = 0.425), an mRS of 0–2 at 90 days (aOR 1.36, CI 0.83–2.21, *p* = 0.223), sICH (aOR 0.39, CI 0.12–1.23, *p* = 0.108), and mortality (aOR 0.49, CI 0.16–1.44, *p* = 0.193). After propensity score matching, the results were consistent with the primary analysis.

**Conclusion:**

Compared to non-RESET, patients treated with RESET showed increased FPE incidence and significantly decreased puncture-to-reperfusion time. RESET was proven to be safe and effective in enhancing reperfusion for LVO patients receiving EVT with underlying ICAS.

## 1 Introduction

In recent years, numerous randomized controlled trials (RCTs) have provided Class I evidence for the safety and efficacy of endovascular treatment (EVT) in acute ischemic stroke (AIS) caused by large vessel occlusion (LVO) (1). Rapid, complete recanalization of these occluded vessels is critical in acute stroke settings, leading to beneficial clinical outcomes especially when successful recanalization is attained in a single or few thrombectomy attempts (2, 3). Since their inception, EVT devices have significantly advanced. Increasing evidence suggests the effectiveness and safety of stent-retrievers, used in combination with other devices, for managing AIS using various techniques (4–12).

Distal intracranial catheters (DICs) are widely utilized for continuous clot aspiration, owing to their exceptional efficacy in attaining high recanalization rates and reducing incidences of thrombi dislodgement (13). Previous studies have predominantly concentrated on advancing the DIC's distal end to the carotid siphon level to achieve thrombus proximity. Nevertheless, the optimal and accurate positioning of DICs prior to EVT has not been the focus of these studies. Previous thrombectomy techniques have been associated with thrombus fragmentation (4, 5) likely due to the substantial distance between the clot's proximal section and the DIC's distal end, through which the stent retriever navigates. Positioning the DIC nearer to the thrombus and concurrently performing stent retrieval and aspiration could offer benefits. Modified techniques such as stent retriever-assisted vacuum-locked extraction (SAVE) and stent-retrieving into an aspiration catheter with proximal balloon (ASAP) technique (6, 12) have been utilized for complete and rapid recanalization. However, these techniques need to be considered and applied in the context of intracranial atherosclerotic stenosis (ICAS), which demonstrates a prevalence of 46.6% in the Asian population (14) and has emerged as a predominant cause of AIS. Additionally, ICAS lesions exhibit a propensity for reocclusion (14), and no prior techniques have been specifically adapted for patients with underlying ICAS. As a result, we developed a modified retrieval method involving complete unfolding of the stent retriever, followed by advancing the DIC or microcatheter to partially re-sheath the stent retriever, creating a tapered configuration. This technique shortens the travel distance for the thrombi-embedded stent retriever and embeds the thrombi more tightly within the strut, preventing clot escape. Herein, we present our experience employing the retrograde semi-retrieval technique (RESET) for EVT in ICAS patients compared to those treated without RESET.

## 2 Materials and methods

### 2.1 Study population

We conducted a retrospective analysis of consecutive patients admitted to Tianjin Huanhu Hospital from January 2018 to December 2022, who underwent EVT with underlying ICAS. All patient data were extracted from the triage of patients with acute ischemic stroke due to large vessel occlusions (TRACK-LVO) registry. A certified neurologist evaluated the National Institute of Health Stroke Scale (NIHSS), modified Rankin scale (mRS), and Alberta Stroke Program Early Computed Tomography (ASPECT) scores during the hospitalization. The diagnosis of ischemic stroke was confirmed using non-contrast computed tomography (NCCT) or magnetic resonance imaging (MRI). The inclusion criteria were as follows: ICAS patients with LVO in the anterior circulation; patients presenting within 24 h from the last known well (LKW) time; patients with a baseline NIHSS score ≥ 6; and patients aged 18 years and above. Patients matching the following criteria were excluded: detection of any form of intracranial hemorrhage on CT or MRI; presence of tandem lesions; and loss to follow-up. Patients were categorized into two groups—those treated with RESET and those without—based on the treating physicians' discretion.

### 2.2 Study data

We examined the following variables: (1) preoperative characteristics such as age, gender, occlusion site, CT/diffusion-weighted imaging (DWI) ASPECT score, NIHSS score, onset-to-door time, onset-to-puncture time, and cardiovascular risk factors; (2) procedure-specific attributes including puncture-to-reperfusion time, number of thrombectomy pass, rescue therapy used in EVT (for example, angioplasty, stenting, intra-arterial thrombolysis, intra-arterial IIb/IIIa inhibitor or some combination), and modified thrombolysis in the cerebral infarction (mTICI) score. Patients with truncal-type occlusions were classified as having underlying ICAS. The diagnosis of truncal-type occlusion was primarily based on evaluations using computed tomography angiography (CTA). If the bifurcation site was distinctly visible on the CTA, it was categorized as truncal-type occlusion. The occlusion type was further confirmed with DSA during EVT. In instances where digital subtraction angiography (DSA) revealed all major branches and their bifurcation site to be clearly observable beyond the occlusion site, the LVO was classified as truncal-type occlusion. Distal confirmation could be achieved either through the collateral flow of the anterior communicating artery, observed on the contralateral internal cerebral artery angiogram or subsequent to minimal recanalization of the occlusion site post the placement of the stent retriever across the site of occlusion (15, 16). The level of intracranial arterial stenosis was determined using the Warfarin–Aspirin Symptomatic Intracranial Disease criteria (17).

### 2.3 Study outcomes

The primary outcome was the first pass effect (FPE), which is defined as the successful removal of a thrombus in a single thrombectomy device pass, which leads to near-complete or complete revascularization. Secondary outcomes included successful reperfusion (mTICI score ≥ 2b at the end of the procedure), functional independence at 90 days follow-up [modified Rankin Scale (mRS) 0–2], all-cause mortality, and symptomatic intracerebral hemorrhage (sICH). The mRS score was assessed by a stroke neurologist during a 3-month follow-up visit or, in the event a clinical visit was impossible, *via* a telephone interview. sICH was classified according to the European Collaborative Acute Stroke Study (ECASS) II criteria, defined as any intracranial hemorrhage with an increase in the NIHSS score of ≥4 within 24 h or resultant death (18).

### 2.4 Endovascular intervention protocol

Endovascular treatment procedures were executed by two interventional neuroradiologists, each with over 5 years of neurovascular intervention experience. Written informed consent was procured from the patient's family members prior to the initiation of the procedure.

Upon placement of the guiding and intermediate catheters in the cervical segment of the internal carotid artery, the microguidewire was used to traverse the occlusion site, and the microcatheter, under the guidance of the microguidewire, reached the distal end of the occluded vessel for intraluminal angiography. This procedure confirmed the presence of the microcatheter in the true lumen of the distal occlusion vessel, enabling the deployment of the thrombectomy stent. Subsequent angiography was performed to identify the site of occlusion, the thrombus burden, and the potential stroke etiology.

If the presence of ICAS was confirmed, a semi-retrieval stent technique was employed. The stent retriever was initially semi-deployed by advancing the DIC. Nonetheless, in certain scenarios, significant stenosis might impede the re-sheathing of the stent retriever by the DIC. In cases of significant resistance encountered while advancing the DIC due to underlying ICAS, a microcatheter was used to re-sheath the stent. Subsequently, the DIC was held in place, and the stent was withdrawn under negative pressure. The DIC was retained in a distal position to facilitate potential rescue therapy.

If the distal thrombus could not be removed due to vascular stenosis, a balloon, guided by a microwire, was used to dilate the stenotic region before next thrombectomy. To prevent downstream thrombus displacement following post-dilation stenosis relief, the DIC maintained continuous negative pressure. After thrombectomy, if residual severe stenosis (≥70%), a mTICI score of <2b or a tendency for re-occlusion were observed, rescue measures such as balloon angioplasty, the preferred option, or stent implantation as an alternative under specific conditions (such as blood vessel dissection or elastic recoiling of the ICAS lesion), were undertaken.

**Figure 1:**
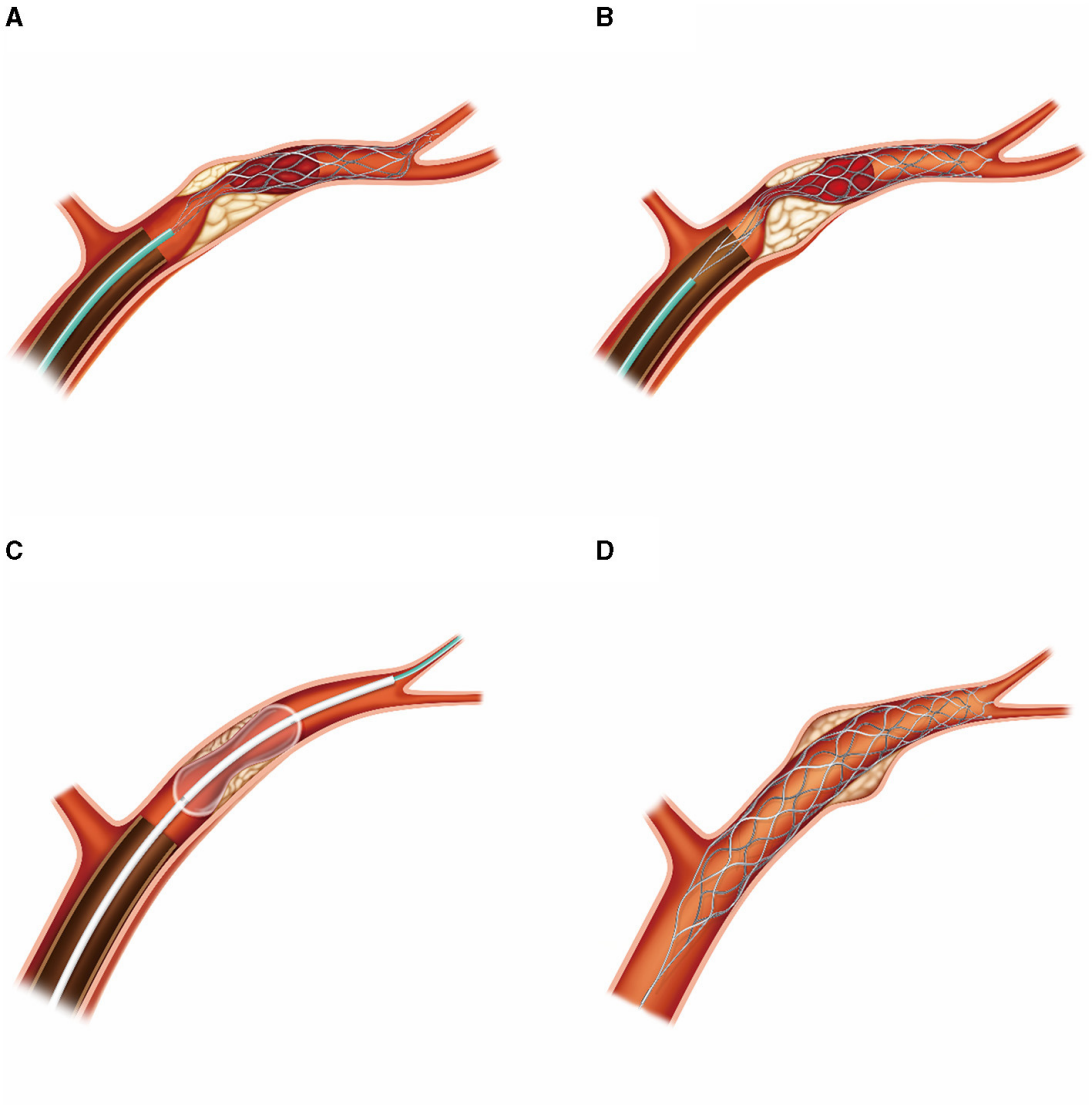
Hand-drawn illustration of the retrograde semi-retrieval technique in patients with ICAS. **(A)** Stent-retriever was fully deployed across the thrombus, with the DIC advanced until its tip was over the ACA's orifice. **(B)** Microcatheter was maintained in position to re-sheath the stent retriever in case of failure of re-sheath process by DIC. The DIC was advanced to attain proximity to the thrombus and held in place to generate maximum negative pressure during stent retriever withdrawal. **(C)** Rescue therapy, such as stent-retriever implantation or balloon dilation, was carried out on residual stenosis as required through the DIC. **(D)** In cases where compromised blood flow persisted, or vascular dissection occurred post-balloon dilation, a Solitaire stent was implanted. ACA, anterior cerebral artery.

**Figure 2:**
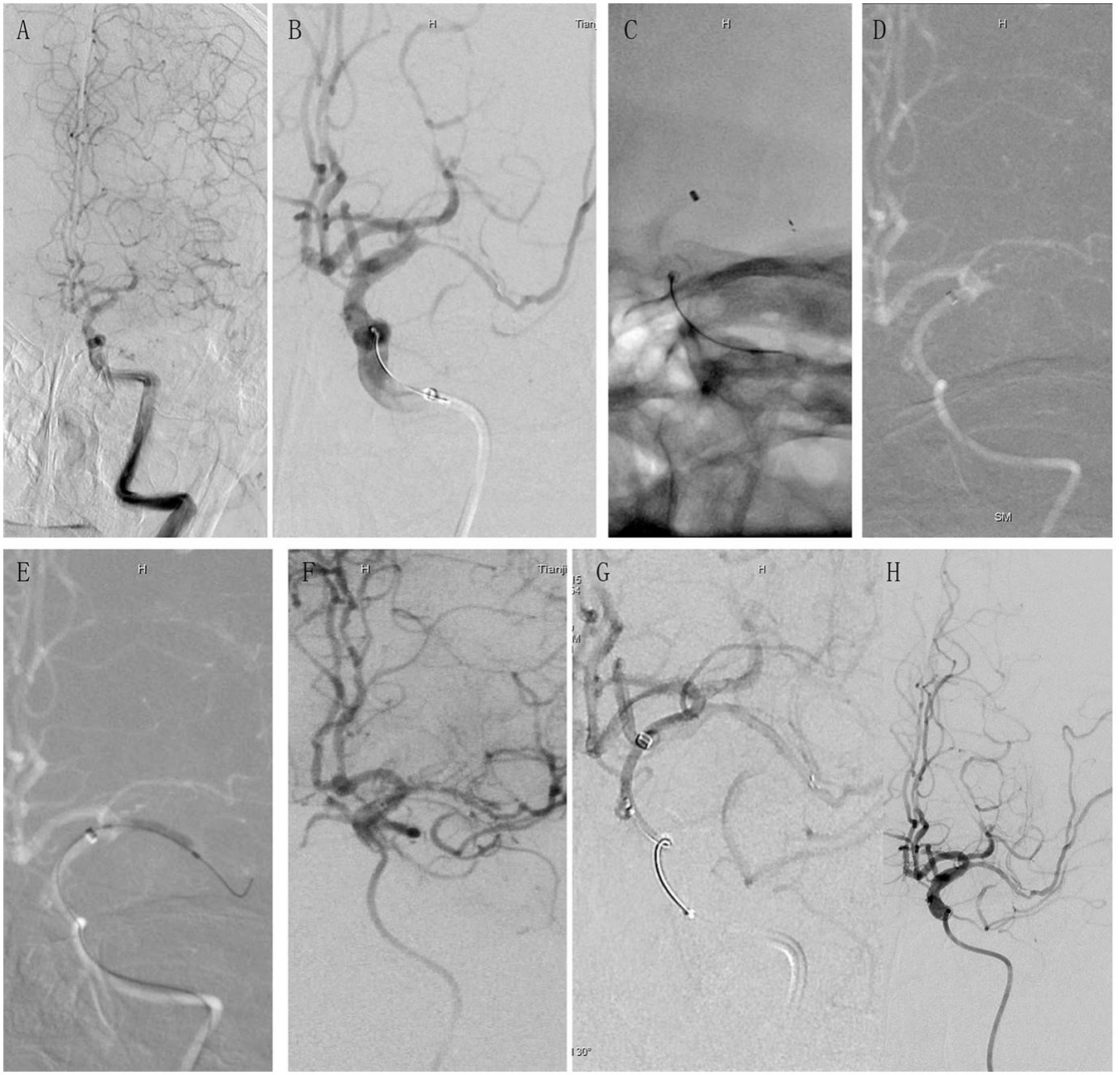
A case presentation showcasing the application of the retrograde semi-retrieval technique in a real-world clinical setting for a patient with ICAS. A 67-year-old male with a medical history of hypertension and diabetes, and no indication of atrial fibrillation, presented to the emergency room with sudden right limb numbness and hemiplegia, which had started 10 h before admission. His NIHSS score upon admission was 9, clinically suggestive of AIS due to LVO with underlying ICAS. **(A)** Preoperative angiography revealed occlusion in the left MCA. The bilateral tissue regions supplied by the ACA were sustained by one dominant ACA, and the ICA was tortuous. **(B)** DIC was advanced to the petrous portion of the ICA and a Solitaire FR stent was deployed. Severe stenosis observed in the MCA on angiography further substantiated the ICAS diagnosis. **(C)** DIC was advanced beyond the ophthalmic artery (OA) origin, up to the C7 segment of the ICA. However, it was unable to progress further into the MCA. The stent was semi-retrieved using the microcatheter, following which the microcatheter and Solitaire FR stent were withdrawn as a single unit. **(D)** Angiography postretrieval of the Solitaire FR demonstrated unrecanalized MCA. **(E)** Rescue balloon angioplasty was initiated *via* the unwithdrawn DIC. **(F)** Although the MCA recanalized following angioplasty, potential indicators of vascular dissection were discerned. **(G)** The Solitaire FR stent was redeployed, and subsequent angiography revealed no signs of dissection. **(H)** The removal of the Solitaire FR stent resulted in good vascular patency, as per the angiography. NIHSS, National Institute of Health Stroke Scale; AIS, acute ischemic stroke; LVO, large vessel occlusion; ICAS, intracranial atherosclerotic stenosis; ACA, anterior cerebral artery; MCA, middle cerebral artery; ICA, internal carotid artery.

Following each rescue therapy, manual aspiration *via* the DIC was applied for 30 s under negative pressure. If preoperative chronic stenosis, intraoperative endothelial injury, or *in-situ* thrombosis leading to vascular re-occlusion were detected, intraoperative administration of tirofiban was performed. A comprehensive overview of the aforementioned technique is shown in Figures 1, 2.

### 2.5 Statistical analysis

The comparative analysis involved the baseline characteristics and clinical outcomes between the two groups. Appropriate statistical tests were utilized to analyze the data: categorical and binary variables were examined using the chi-square test or Fisher's exact test as necessary, while continuous variables were analyzed with the Mann–Whitney *U*-test. Continuous variables are represented as mean ± SD for a normal distribution and as a median with IQR for a skewed distribution. Percentages along with their corresponding 95% CIs were used to present categorical variables. Statistical comparisons of baseline characteristics and study outcomes were performed for patients treated with and without RESET. Multivariable regression analysis was subsequently performed, with adjustments made for clinically relevant variables: age, gender, baseline stroke severity (as per the NIHSS), clot location, and presentation time from LKW. Sensitivity analysis was conducted through propensity score matching (PSM), for which the propensity score was computed based on logistic regression models while controlling for the aforementioned covariates. R software (version 4.3.2 for Windows) was employed for all statistical analyses. A *p*-value of <0.05 was considered as statistically significant.

## 3 Results

### 3.1 Baseline characteristics of study population

**Figure 3:**
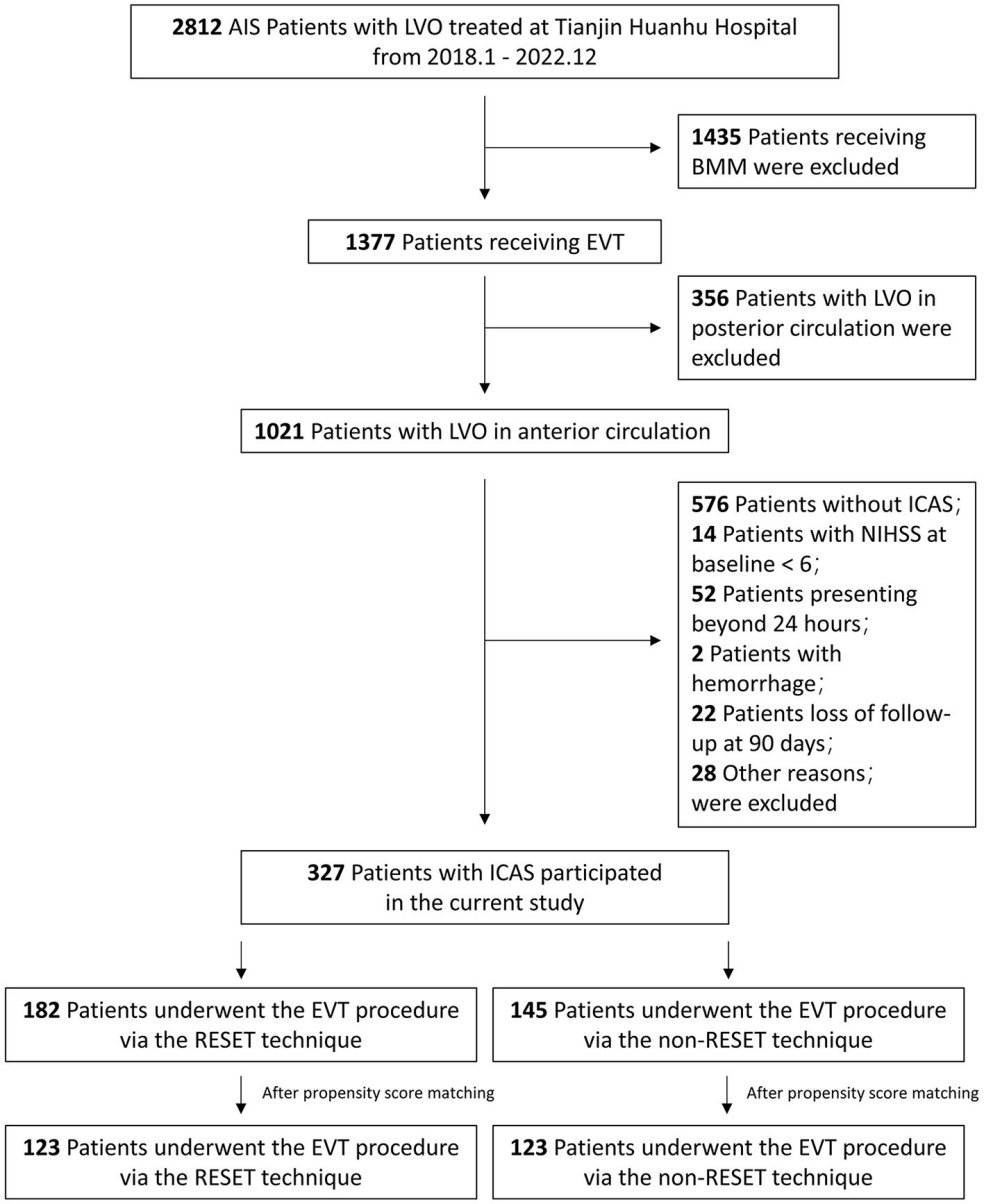
Study flow chart. AIS, acute ischemic stroke; BMM, best medical management; EVT, endovascular thrombectomy; ICAS, intracranial atherosclerotic stenosis; LVO, large vessel occlusion; NIHSS, National Institute of Health stroke scale; RESET, Retrograde Semi-Retrieval Technique.

Our study period analysis involved a total of 2,812 AIS patients registered in the TRACK-LVO registry. Among these, we included 327 patients with underlying ICAS (182 treated with the RESET technique and 145 with non-RESET) meeting the requisite inclusion and exclusion criteria for the final analysis (see Figure 3).

When compared to patients treated with EVT using the non-RESET method, those treated with EVT using the RESET technique displayed a lower likelihood of being male (66.5% in RESET vs. 80.0% in non-RESET, *p* = 0.009), and a trend toward older age (mean age 61.71 ± 12.10 years vs. 59.43 ± 12.12 years, *p* = 0.093) and a higher baseline stroke severity as measured by NIHSS [median score 11.0 (9.0–15.0) vs. 11.0 (9.0–13.0), *p* = 0.062]. The baseline CT ASPECTS [median score 9.0 (8.0–10.0) in both groups, *p* = 0.093] was not significantly different between the two groups. However, the DWI-ASPECTS score was significantly lower in the non-RESET group compared to the RESET group [median 7.0 (6.0–8.0) vs. 7.0 (6.5–8.0), *p* = 0.008]. No significant differences were noted in the remaining clinically relevant baseline characteristics.

In terms of cardiovascular risk factors, RESET-treated patients were more likely to have hypertension (76.9% in RESET vs. 63.2% in non-RESET, *p* = 0.007) and previous ischemic stroke events (18.7% in RESET vs. 7.6% in non-RESET, *p* = 0.004), but a significantly reduced incidence of atrial fibrillation (2.7% in RESET vs. 23.3% in non-RESET, *p* < 0.001). Table 1 summarizes baseline information of the two groups.

**Table 1:** Comparison of baseline characteristics between two groups.

**Variable**	**RESET group (*N* = 182)**	**Non-RESET group (*N* = 145)**	***P***
Age, mean (SD), years	61.71 (12.10)	59.43 (12.12)	0.093
Males, *n* (%)	121 (66.5)	116 (80.0)	0.009
Hypertension, *n* (%)	140 (76.9)	91 (63.2)	0.007
Diabetes mellitus, *n* (%)	37 (25.0)	24 (16.9)	0.113
Coronary artery disease, *n* (%)	22 (12.1)	17 (12.6)	1
Atrial fibrillation, *n* (%)	5 (2.7)	31 (23.3)	<0.001
Dyslipidemia, *n* (%)	63 (34.6)	35 (24.1)	0.052
TIA, *n* (%)	15 (8.2)	12 (8.3)	1
Previous ischemic stroke, *n* (%)	34 (18.7)	11 (7.6)	0.004
Smoking, *n* (%)	78 (42.9)	85 (58.6)	0.005
Alcohol, *n* (%)	59 (32.4)	56 (38.6)	0.247
CT ASPECTS, median (IQR)	9.0 [8.0–10.0]	9.0 [8.0–10.0]	0.093
DWI ASPECTS, median (IQR)	7.0 [6.0–8.0]	7.0 [6.5–8.0]	0.008
NIHSS, median (IQR)	11.0 [9.0–15.0]	11.0 [9.0–13.0]	0.062
Site of occlusion, *n* (%)			0.298
M1	71 (61.7)	116 (67.8)	
M2	16 (13.9)	6 (3.5)	
ICA	28 (24.3)	49 (28.7)	
Others	3 (1.6)	2 (1.4)	

CT, computed tomography; DWI, diffusion-weighted imaging; TIA, transient ischemic attack; ASPECTS, Alberta Stroke Program Early CT Score; NIHSS, National Institute of Health stroke scale.

Data are expressed as medians (IQR) Mean ± SD, and n (%).

### 3.2 Primary outcome of study population

FPE occurred in 38 (20.9%) patients in the RESET group as compared to 17 (11.7%) patients in the non-RESET group (*p* = 0.037). After adjustment, RESET was significantly associated with FPE [adjusted odds ratio (aOR) 2.00, 95% confidence interval (CI) 1.03–3.87, *p* = 0.040] (see Table 2).

**Table 2:** Procedural characteristics and study outcomes between two groups.

**Variable**	**RESET group (*n* = 182)**	**Non-RESET group (*n* = 145)**	***P***
Time from onset-to-door, median (IQR), min	427 [226–544]	449 [277–576]	0.391
Time from door-to-puncture, median (IQR), min	91 [53–120]	91 [45–116]	0.68
Time from puncture-to-recanalization, median (IQR), min	125 [89–144]	136 [93–160]	0.008
Time from onset-to-recanalization, median (IQR), min	529 [459–575]	530 [483–582]	0.221
Intracranial stenting, *n* (%)	15 (8.2)	12 (8.3)	1
Angioplasty alone, *n* (%)	18 (9.9)	14 (9.7)	1
Angioplasty + IIb/IIIa inhibitor, *n* (%)	110 (60.4)	91 (62.8)	0.732
IIb/IIIa inhibitor, *n* (%)	93 (51.1)	78 (53.8)	0.657
Number of techniques, median (IQR), times	1 [1–1]	1 [1–2]	0.11
First pass recanalization, *n* (%)	38 (20.9)	17 (11.7)	0.037
Final reperfusion grade (mTICI), *n* (%)			0.016
Grade 0	1 (0.5)	3 (2.1)	
Grade 1	1 (0.5)	1 (0.7)	
Grade 2a	7 (3.8)	6 (4.1)	
Grade 2b	58 (31.9)	25 (17.2)	
Grade 3	115 (63.2)	110 (75.9)	
mTICI 2b-3, *n* (%)	173 (95.1)	135 (93.1)	0.483
sICH, *n* (%)	6 (3.3)	9 (6.2)	0.288
Any ICH, *n* (%)	22 (12.1)	13 (9.0)	0.472
mRS (0–2), *n* (%)	102 (56.0)	76 (52.4)	0.576
Mortality, *n* (%)	7 (3.8)	9 (6.2)	0.44

mTICI, modified thrombolysis in cerebral infarction; sICH, symptomatic intracranial hemorrhage; mRS, modified Rankin Scale; IA, intra-arterial.

Data are expressed as medians (IQR), Mean ± SD, and n (%).

### 3.3 Secondary outcomes of study population

In the RESET cohort, successful reperfusion was achieved in 173 patients (95.1%) compared to 135 patients (93.1%) in the non-RESET cohort (*p* = 0.483). At the 3-month follow-up, an mRS of 0–2 was observed in 102 patients (56.0%) in the RESET group and in 76 patients (52.4%) in the non-RESET group (*p* = 0.576). The occurrence of sICH was noted in 6 patients (3.3%) from the RESET group and in 9 patients (6.2%) from the non-RESET group (*p* = 0.288). Mortality was less frequently observed in the RESET group, with seven deaths (3.8%) as compared to the non-RESET group which recorded nine deaths (6.2%) (*p* = 0.440). Upon adjustment, there was no significant association between the RESET group and the successful reperfusion (aOR 1.5, CI 0.55–4.06, *p* = 0.425), mRS of 0–2 at 90 days (aOR 1.36, CI 0.83–2.21, *p* = 0.223), incidence of sICH (adjusted OR 0.39, CI 0.12–1.23, *p* = 0.108), and mortality rate (adjusted OR 0.49, CI 0.16–1.44, *p* = 0.193) (see Table 2).

### 3.4 Study outcomes in the PSM population

Following propensity score matching, the cohort was refined to include 123 patients each in the RESET and non-RESET groups. These results were consistent with the primary analysis. Specifically, the RESET technique exhibited a significant association with FPE (aOR 2.85, CI 1.45–5.63, *p* = 0.002) when compared to the non-RESET group. However, no significant differences were observed in successful reperfusion (aOR 2.06, CI 0.59–7.19, *p* = 0.256), mRS of 0–2 at 90 days (aOR 1.34, CI 0.77–2.34, *p* = 0.297), incidence of sICH (aOR 0.46, CI 0.14–1.51, *p* = 0.198), and mortality rate (aOR 0.5, CI 0.15–1.68, *p* = 0.264).

## 4 Discussion

EVT has been demonstrated to be both safe and effective in the treatment of AIS caused by LVO. However, it is imperative to acknowledge that, while efficacious, EVT does not wholly eradicate the risks of morbidity and mortality (1). Prior research studies have suggested that successful recanalization achieved in a single or minimal thrombectomy attempt correlates with improved patient outcomes (2, 3). Nevertheless, complete reperfusion with a single pass is accomplished in <50% of patients, often necessitating multiple thrombectomy endeavors or even supplementary rescue therapy (19, 20). Multiple passes of thrombectomy devices might also extend the procedure duration, inflict harm to arterial endothelial tissue, and potentially impact clinical outcomes negatively (21, 22).

In the context of ongoing innovations in thrombectomy devices, the primary objective of thrombectomy should be to achieve complete revascularization with as few passes as possible. Nevertheless, an optimal EVT technique consensus, particularly in ICAS patients, is yet to be established (23). A prior report suggested the use of ADAPT for the initial attempt due to its association with higher reperfusion rates, increased positive outcomes, and reduced hemorrhagic complications. However, it still results in failed ADAPT in approximately one-third of cases, necessitating rescue therapy (24). Given that previous studies on aspiration techniques rarely included Asian populations (25), which display a substantial prevalence of ICAS, ADAPT may not be an appropriate treatment for severe atherosclerotic stenosis under such conditions. Other techniques, such as the Solumbra technique, continuous aspiration prior to intracranial vascular embolectomy (CAPTIVE) (8), SAVE, ASAP, Aspiration-Retriever technique for stroke (ARTS) (7), guide sheath advancement and aspiration in the distal petrocavernous ICA (GUARD) (26), balloon guide with large bore distal access catheter with dual aspiration with stent-retriever as standard (BADDASS) (11), and proximal balloon occlusion together with direct thrombus aspiration (PROTECT) (9, 10) involve a hybrid approach of aspiration and stent retrieval. However, these techniques' efficacy and safety remain untested in ICAS patients.

Our retrograde semi-retrieval technique offers several advantages over previously mentioned methods. First, it is a straightforward procedure that does not necessitate the use of complex devices such as balloon-guiding catheters or aspiration pumps, which can be financially burdensome for hospitals in many developing nations. Prior methods have employed an 8–9 Fr balloon-guiding catheter to achieve flow arrest; however, in situations with tortuous aortic arches, utilizing a larger balloon-guiding catheter poses a significant challenge. Additionally, the complication rate at the groin puncture site is estimated to be as high as 0.8% (27). Catheters with sheath sizes >8F have been associated with extended hospital stays and elevated mortality rates (28). Second, this method offers greater flexibility, with strategies varying based on whether the patient has an underlying ICAS. It has also been employed for patients without ICAS, as previously reported (29). The aspiration impact can be fine-tuned by adjusting the manual aspiration force and the retraction of the microcatheter (in cases where DIC could semi-retrieve the stent-retriever) (Figure 1). The aspiration catheter remains stationary at the location where the stent-retriever was withdrawn, applying a constant aspiration force to eliminate any potential thrombus fragments. This technique is similar to the ASAP method (12) but does not involve inflating a balloon guide catheter in the cervical ICA. Third, a DIC provides stability in the M1 segment, thereby enabling the swift execution of rescue therapy. This makes RESET an optimal thrombectomy procedure for ICAS patients as most ICAS patients require rescue therapy. In the majority of MCA occlusion cases, the DIC can be easily advanced to the MCA (13), facilitating the rapid exchange of the balloon or stent deployment. Fourth, the DIC is advanced close to the clot or the residual stenotic lesion, maximizing the aspiration force, reducing the clot-retrieval distance, and inhibiting thrombus migration. This simplifies the treatment and subsequent rescue therapies for atherosclerotic stenosis.

A potential concern about the RESET technique is its potential suboptimality when handling high-load thrombus due to the risk of fragment detachment and subsequent distal embolization, despite continuous aspiration. Nonetheless, in patients with ICAS, thrombi are typically smaller in size (30), making the blend of stent retrieval and aspiration generally adequate for successful clot removal. In cases of MCA occlusion, most stenosis typically localizes in the middle of the M1 segment as opposed to the bifurcation, a scenario more commonly observed in embolic strokes. This location is conducive for DIC advancement, facilitating more intimate contact with ICAS lesions, and potentially enabling even high-load thrombus management. In our routine practice, we seldom resort to alternative thrombectomy techniques and infrequently encounter thrombus distal embolization. Therefore, this strategy may serve well in treating patients presenting with ICAS.

In the propensity score-matched population, the FPE rate was achieved in 35 (28.5%) patients using the RESET technique, which is commendable considering the complexity of thrombectomy procedures in ICAS patients. In a single-center study comparing the efficacy of ADAPT and Solumbra techniques in ICAS patients, FPE was observed in 9 (16.1%) patients using ADAPT and 12 (25.0%) patients using the Solumbra technique (24). This discrepancy in FPE attainment can be attributed to several factors. First, inherent characteristics of RESET, such as the more distal positioning of the DIC, may account for some of this difference. The distal end of the DIC is closer to the ICAS lesion, which maximizes the aspiration force. Second, the aspiration starts while advancing the DIC to semi-retrieve the stent-retriever. Third, the semi-retrieval process brings the stent and the embedded thrombus into close contact, causing the thrombus to compact, which aids the retrieval process. The combined effect of a robust aspiration force, adequate aspiration during the stent semi-retrieval process and increased contact between the stent and thrombus collectively contribute to an enhanced FPE.

A significant reduction in the time from puncture to reperfusion was also noticed in patients treated with RESET. Previous research has established a link between decreased puncture-to-reperfusion time and improved functional outcomes (31, 32). The use of RESET for ICAS patients may enhance functional independence, as indicated by our findings showing a numerically higher proportion of patients achieving functional independence with RESET as the primary thrombectomy technique (RESET 56.0% vs. non-RESET 52.4%). The reduction in puncture-to-reperfusion time may be attributed to this technique's ability to swiftly facilitate rescue therapy. Prior research has demonstrated that both non-compliant balloons and intracranial stents are effective and safe for mechanical thrombectomy in acute ischemic stroke patients with ICAS and emergent LVO (33). However, in earlier studies, the rescue therapy procedure was complex and time-consuming. In this study, to rapidly reach the thrombus, the DIC was advanced as far as possible to improve access for balloon or stent delivery (Figure 2). Given that ICAS patients often present with severe stenotic lesions or tortuous intracranial blood vessels, making DIC advancement challenging, we utilize coaxial techniques and the stent's anchoring force to quickly deliver the DIC.

In our propensity score-matched population, we reported a sICH rate of 4.1% and a mortality rate of 4.1%. When compared to a previous meta-analysis that reported pooled rates of sICH and mortality at 5.5 and 20.2%, respectively, our study suggests that the RESET technique may be relatively safer (34). Numerous studies have indicated that more than three thrombectomy passes can be associated with intracerebral hemorrhage (35). In our study, upon confirmation of ICAS, we typically proceed directly to rescue therapy. Consequently, the number of thrombectomy passes is often limited to no more than two passes, assisted by aspiration. Furthermore, following each rescue therapy, we initiate aspiration to prevent the dispersion of emboli during the procedure. Finally, the semi-retrieval of the stent leads to a denser thrombus and a more secure adhesion between the stent and thrombus, further reducing the risk of thrombus embolization. These factors could potentially decrease the likelihood of thrombus migration and may elucidate the safety profile of the RESET technique.

The most effective thrombectomy technique for ICAS patients remains a subject of ongoing debate. While some neurointerventionists posit that stent thrombectomy surpasses aspiration in efficacy, the RESET method merges the strong suits of both techniques, thereby offering improved efficiency and safety. Therefore, the retrograde semi-retrieval technique merits consideration as a first-line treatment for patients with LVO and concomitant underlying ICAS.

This study has several limitations, including its retrospective design, single-center scope, and the exclusive enrollment of Chinese patients. Furthermore, selection bias may be present since patients with atrial fibrillation, who are presumed to have large-load thrombus, led treating physicians to favor non-RESET techniques. Future prospective multicenter studies are necessary to more comprehensively assess the potential advantages of the RESET technique over other methods in patients with AIS due to LVO with underlying ICAS.

In conclusion, the RESET technique is safe, effective, and applicable for patients with acute LVO caused by ICAS.

## Data availability statement

The raw data supporting the conclusions of this article will be made available by the authors, without undue reservation.

## Ethics statement

Ethical review and approval was not required for the study on human participants in accordance with the local legislation and institutional requirements. Written informed consent from the patients/participants or patients/participants' legal guardian/next of kin was not required to participate in this study in accordance with the national legislation and the institutional requirements.

## Author contributions

WW: Data curation, Project administration, Writing—original draft. YX: Data curation, Formal analysis, Writing—original draft. BZ: Methodology, Project administration, Writing—review & editing. SL: Writing—review & editing, Data curation. ZM: Writing—original draft & editing, Visualization, Resources. SW: Writing—original draft, Validation, Investigation. PZ: Writing—review & editing, Methodology, Conceptualization, Formal analysis. MW: Methodology, Conceptualization, Writing—review & editing.
